# Beneficial effects of combination therapy with testosterone and hydrogen sulfide by reducing oxidative stress and apoptosis: Rat experimental varicocele model

**DOI:** 10.18502/ijrm.v20i11.12362

**Published:** 2022-12-10

**Authors:** Anahid Shafie, Farzaneh Kianian, Ghorbangol Ashabi, Mehri Kadkhodaee, Mina Ranjbaran, Mahdi Hajiaqaei, Keivan Lorian, Arash Abdi, Behjat Seifi

**Affiliations:** ^1^Department of Physiology, Faculty of Medicine, Tehran University of Medical Science, Tehran, Iran.; ^2^Research and Clinical Center for Infertility, Yazd Reproductive Sciences Institute, Shahid Sadoughi University of Medical Sciences, Yazd, Iran.

**Keywords:** Apoptosis genes, Hydrogen sulfide, Oxidative stress, Sperm count, Testosterone, Varicocele.

## Abstract

**Background:**

Despite the effectiveness of testosterone therapy in conditions associated with testosterone deficiency, including varicocele, several dose-dependent side effects limit the clinical use of testosterone therapy. Hydrogen sulfide, a toxic gas in high concentrations but a beneficial molecule in low concentrations, acts as both a major effector and an important inducer of testosterone.

**Objective:**

This study investigated whether a subeffective dose of testosterone combined with a subeffective dose of hydrogen sulfide donor sodium hydrosulfide (NaHS) can be effective in an experimental varicocele model through a possible additive effect.

**Materials and Methods:**

Thirty Wistar rats weighing 200-250 gr were divided into 5 groups as (n = 6/each): sham, varicocele, testosterone (200 µg/kg, 5 times per wk for 4 consecutive weeks), NaHS (15 μmol/L, daily for 4 consecutive wk) and testosterone + NaHS (200 µg/kg, 5 times per wk + 15 μmol/L, daily, both for 4 consecutive wk). All animals, except in the sham group, underwent varicocele induction.

**Results:**

The coadministration of testosterone and NaHS significantly increased serum testosterone (10.23 ± 0.95, p = 0.01), testicular H_2_S levels (608.94 ± 21.09, p < 0.001), and testicular superoxide dismutase activity (66.14 ± 1.56, p < 0.001), decreased malondialdehyde levels (0.77 ± 0.52, p < 0.001), and B-cell lymphoma 2-associated X protein to B-cell lymphoma 2 (0.16 ± 0.01, p < 0.001) protein expression ratio in the testicular tissues and improved sperm parameters and testicular histopathology compared to the varicocele group.

**Conclusion:**

The combination therapy of subeffective doses of testosterone and NaHS can attenuate the varicocele-induced damages by reducing testicular oxidative stress and apoptosis and thus can be considered an effective approach with fewer side effects.

## 1. Introduction

Male fertility requires the continuous production of an adequate number of motile and morphologically normal spermatozoa (1). This process strongly depends on testosterone produced by the Leydig cells in the testes; thus, testosterone deficiency leads to impaired male fertility (2). In this regard, many studies have reported testosterone deficiency in patients with varicocele. The abnormal dilatation of the pampiniform plexus, based on the world health organization, is the leading cause of infertility in men (3-5). Several studies have shown that varicocele is associated with decreased sperm count, motility, morphology, and semen volume (6, 7). To date, surgical varicocelectomy is considered as the gold standard treatment for varicocele. Nevertheless, besides having several side effects, the association between varicocelectomy and improved testosterone levels remains controversial (8).

Testosterone therapy has long been the main approach for solving the testosterone deficiency problem. Administration of testosterone in appropriate doses stimulates spermatogenesis, increases sperm concentration and motility, and ameliorates inflammation, oxidative stress, and apoptosis by suppressing testicular damage (9). However, despite its effectiveness and simplicity, the clinical use of testosterone therapy has been bounded by reason of dose-dependent numerous side effects such as prostate cancer and cardiovascular risks (10). In addition, long-term exogenous administration of testosterone suppresses the hypothalamic-pituitary-gonadal axis, which may partially or completely stop spermatogenesis by reducing follicle-stimulating hormone and luteinizing hormone (11). Therefore, it is essential to investigate how to take advantage of testosterone therapy while at the same time reducing its side effects. An interesting result of research on the action mechanism of testosterone is that the beneficial effects of this hormone are mediated, at least in part, by hydrogen sulfide (H_2_S), being toxic in high concentrations but acts as an endogenous gaseous signaling molecule in low concentrations (12, 13). On the other hand, it has been shown that H_2_S can increase testosterone levels (14). These findings drew our attention to H_2_S to see if a low (subeffective) dose of testosterone combined with a subeffective dose of H_2_S can be effective in treating varicocele through a possible additive effect. Furthermore, given the involvement of different pathological mechanisms such as oxidative stress and apoptosis in the pathophysiology of varicocele, the fact that H_2_S, similar to testosterone, has various beneficial biological properties, including antioxidant and antiapoptotic activities prompted us further to choose H_2_S for this combination therapy (1, 15).

To our knowledge, the therapeutic effects of a subeffective dose of testosterone in combination with a subeffective dose of the H_2_S donor sodium hydrosulfide (NaHS) on infertility disorders have not been studied so far, the present study aims to investigate the beneficial effects of the combined administration of subeffective doses of testosterone and NaHS on a rat model of varicocele along with the underlying mechanisms.

## 2. Materials and Methods

### Animals

For this experimental study, 8-10 wk-old, outbred albino Wistar rats weighing 200-250 gr were obtained from the Department of Physiology of Tehran University of Medical Sciences, Tehran, Iran. Animals were maintained in an air-conditioned room at 22 ± 2 C under a 12 hr light/dark cycle with access to food and water ad libitum. Three rats were kept within each cage. Deep sedation was induced to prevent pain, and antibiotic tetracycline was used at the surgical site to reduce infection (16).

### Varicocele induction

The varicocele model was induced by partial ligation of the left renal vein (17, 18). Briefly, rats were intraperitoneally anesthetized with 100 mg/kg ketamine (Rotexmedica, Germany) and 10 mg/kg xylazine (Sigma, USA). A 4-0 silk thread was placed around the left renal vein over a 0.7 mm needle and tied snugly; then the needle was removed. Sham rats were subjected to the same procedures, except that the left renal vein was not tied.

### Dose-response

To select a subeffective dose of testosterone (Padgin Teb Co, Iran), 5 wk after the induction of varicocele, rats were subcutaneously injected with testosterone at doses of 100 (19), 200, and 400 µg/kg (20), 5 times per wk for 4 consecutive weeks, and then sperm parameters such as count, motility, viability, and morphology were assessed. However, the treatment duration was chosen according to the spermatogenesis period in rats. Our results showed significant improvements in all these parameters in rats that received 400 µg/kg testosterone, but not in 100 and 200 µg/kg testosterone (data not shown). On the other hand, to select a subeffective dose of NaHS (Merck, Germany), 5 wk after the induction of varicocele, other rats received 15 and 30 μmol/L of NaHS (21) in drinking water daily for 4 consecutive weeks, and then mentioned sperm parameters were evaluated. Our findings revealed marked improvements in all these parameters in rats that received 30 μmol/L NaHS but not 15 μmol/L NaHS. Therefore, 200 µg/kg testosterone and 15 μmol/L NaHS as half of the effective dose were selected as the subeffective doses for further experiments.

### Experimental design

Thirty rats were divided randomly into 5 groups (n = 6) as in previous work in our laboratory: (1) sham, (2) varicocele, (3) testosterone (200 µg/kg, 5 times per wk for 4 consecutive weeks), (4) NaHS (15 μmol/L, daily for 4 wk), and (5) testosterone + NaHS (200 µg/kg, 5 times per wk + 15 μmol/L, daily, both for 4 wk). Five wk after varicocele induction, animals in all treatment groups were treated. Eight wk after varicocele induction, serum samples were collected to measure testosterone levels. Then, the left caudal epididymis was separated, placed in a petri dish containing 1 ml of 37 C sperm media (Ham's F-10, Thermo Fisher Scientific, USA), and cut into pieces to remove the spermatozoa. Then, the sperm samples were placed in the incubator at 37 C. After 15 min, sperm motility, viability, count, and morphology were assessed. Testicular tissues were removed and washed in cold normal saline. The left testes were kept at -80 C to evaluate malondialdehyde (MDA), H_2_S levels, Bcl-2-associated X protein (Bax), and B-cell lymphoma 2 (Bcl-2) protein expression. A part of the right testes was stored at -80 C to determine superoxide dismutase (SOD) activity, and another part of these tissues was fixed in 10% formalin for histopathological study.

### Measurement of serum testosterone levels

Serum testosterone levels were measured by enzyme-linked immunosorbent assay. Before starting the experiment, all reagents, standard diluents, control, and test samples were placed in the laboratory to reach room temperature. Then all procedures were carried out based on the manufacturer's instructions (Padgin Teb Co, Iran). In this test, the quantitative sandwich enzyme immunoassay technique was used. The reactions were quantified by reading the optical density at 450 nm by an enzyme-linked immunosorbent assay reader.

###  Assessment of testicular histopathology

After fixation in 10% formalin (Merck, Germany), testicular tissues were embedded in paraffin, sectioned, and stained with hematoxylin-eosin solution (Inocolon, Iran). The tissue sections were then evaluated for pathological changes in the seminiferous tubules, basement membranes, and surrounding interstitial tissues using a light microscope at × 400 magnification.

### Assessment of sperm count, motility, viability, and morphology

For sperm count, 500 µL of sperm suspension was mixed with 1,200 µL of 20% formalin, and the mixture (10 µL) was then placed on a hemocytometer. In 5 microscopic fields, sperm heads were counted using a light microscope at × 400 magnification (22). For sperm motility, sperm suspension (10 µL) was placed on a 37 C glass slide, and then the motility of 200 spermatozoa was evaluated using a light microscope at × 400 magnification. The evaluation of progressive motile spermatozoa, non-progressive motile spermatozoa and non-motile spermatozoa were performed according to the World Health Organization criteria (23). For sperm viability, 1% eosin and 10% nigrosin (Inocolon, Iran) were separately prepared. 1% eosin (2 volumes) was added to sperm suspension (one volume). After 30 sec, an equal volume of 10% nigrosin was added to the mixture, and then a thin smear was made on a 37 C glass slide. The viability of 100 spermatozoa was evaluated using a light microscope at × 1000 magnification. Viable spermatozoa remained colorless, whereas dead spermatozoa stained pink. For sperm morphology, the eosin-nigrosin staining was used. Eosin and nigrosin (10 µL) were added to sperm suspension (50 µL) on a glass slide, and the smear was incubated for 60 min at room temperature. The morphology of 100 spermatozoa was evaluated using a light microscope at × 1000 magnification (24, 25). The hook-shaped head was considered normal, while the round head, pinhead, double head, amorphous head, bent neck, asymmetrical neck, excess residual cytoplasm more than one-third head, double tail, bent tail, coiled tail, and short tail were considered abnormal (23).

### Morphometric assessment of the seminiferous tubules

In the testicular sections stained with hematoxylin-eosin stain, the tubular diameter, area, and thickness of the seminiferous epithelium of the seminiferous tubules with rounded contours in 10 sections per animal were examined using Motic Image Plus 2.0 ML software. The diameter was defined as the average of the 2 parallel tangent lines on the outer edge of the tubule. The area was determined with the following formula: π (D/2)^2^. The thickness was calculated as the average of the 4 quadrants of the tubule (90 , 180 , 270 , and 360 ) (24).

###  Measurement of testicular H_2_S levels

Fifty mg of testicular tissues was homogenized in 500 µl of phosphate-buffered saline. After incubating the homogenates with L-cysteine (Sigma, USA), pyridoxal phosphate (Sigma, USA), and normal saline for thirty min at 37 C, trichloroacetic acid (Sigma, USA) and zinc acetate (Merck, Germany) were added. Fifteen min after adding N, N-dimethyl-p-phenylenediamine sulfate (Sigma, USA) and ferric chloride (Sigma, USA), the absorbance of aliquots was measured at 660 nm by a microplate reader (26).

### Assessment of the number of the Sertoli cells and spermatogonia

In hematoxylin-eosin-stained testicular sections, the Sertoli cells and spermatogonia in 50 seminiferous tubules per animal were counted using a light microscope at × 400 magnification.

### Measurement of testicular MDA levels

MDA levels of testicular tissues were measured using the Esterbauer and Cheeseman method (27). The testicular tissue was mixed with 10% trichloroacetic acid (Sigma, USA) based on this method. After centrifugation of the mixture at 3000 g for 15 min at 4 C, the supernatant was separated and reacted with thiobarbituric acid (Sigma, USA) in 100 C water for 15 min. The reaction of MDA with thiobarbituric acid produces a pink pigment that has a maximum absorption at 532 nm (28).

### Measurement of testicular SOD activity

SOD activity of testicular tissues was evaluated using the Nasdox ${}^{\text{TM}}$ assay kit according to the manufacturer's protocol (Navand Salamat, Iran). Furthermore, 50 μL of the samples were blended with reagent 1 (200 μL), and reagent 2 (50 μL) was poured into a microplate. After 5 min of incubation, the optical density was determined at 405 nm by a spectrophotometer (29).

### Testicular protein extraction and western blotting

Testicular tissues were homogenized in lysis buffer and centrifuged at 13,000 g for 15 min at 4 C. Then, protein concentrations in cell lysates were measured according to the Bradford method (30). Proteins (20 mg each) were electrophoresed in 12% SDS-PAGE and blotted on a polyvinylidene difluoride membrane. Anti-Bax and anti-Bcl-2 antibodies (Cell Signaling Technology, MA, USA) were used for membrane incubation and probed with horseradish peroxidase-conjugated secondary antibody (Cell Signaling Technology, MA, USA) in the next step. The reactive bands were distinguished by a chemiluminescence detection system, and Image J software was used for quantification of the blots. For normalization, the same polyvinylidene difluoride membrane was re-blotted with an anti-β-actin antibody (Cell Signaling Technology, MA, USA).

### Ethical considerations 

All experiments, including working with laboratory animals, were approved by the Ethics Committee of Tehran University of Medical Sciences, Tehran, Iran (Code: IR.TUMS.MEDICINE.REC.1398.760).

### Statistical analysis

Data are presented as mean ± standard error of the mean (SEM). A comparison among groups was performed by one-way analysis of variance (ANOVA) and Tukey's test. P-values < 0.05 were considered significant. All statistical analyses were performed using SPSS software (SPSS, version 22, Chicago, IL, USA).

## 3. Results

### Effects of subeffective doses of testosterone and NaHS alone or in combination on serum testosterone and testicular H_2_S levels

**Figure 1:**
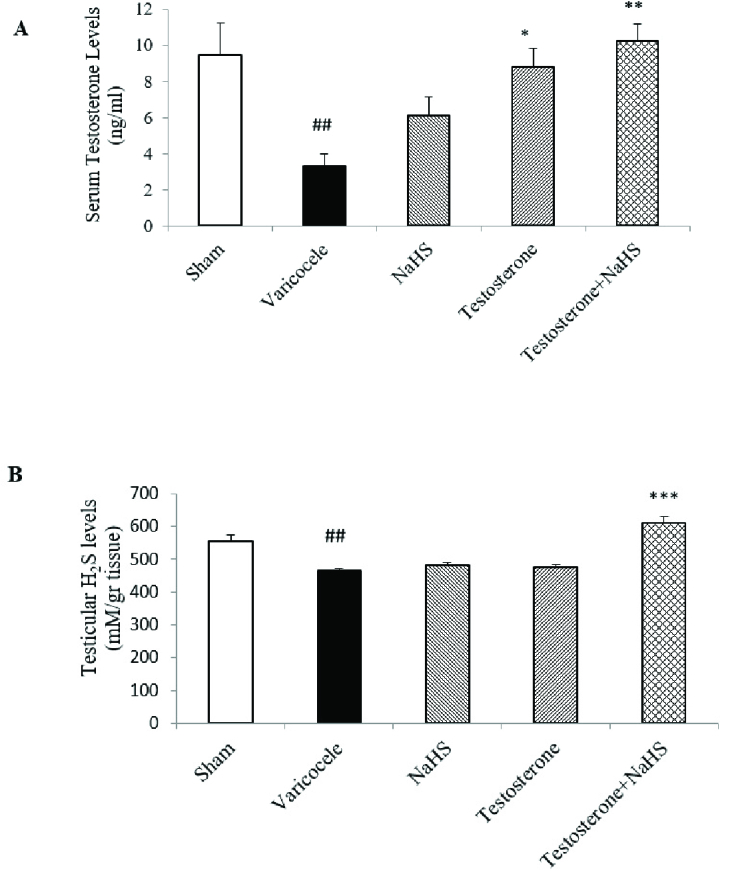
Changes in serum testosterone (A) and testicular H_2_S (B) levels in different groups. A comparison among groups was performed by one-way analysis of variance (ANOVA) and Tukey's test. Data are expressed as Mean ± standard error of the mean (SEM). ${}^{\#\#}$ P < 0.01 vs. the sham group. *P < 0.05 vs. the varicocele group **P < 0.01 vs. the varicocele group. ***P < 0.001 vs. the varicocele group.

The varicocele induction in rats significantly decreased serum testosterone levels compared with the sham group (p = 0.02, Figure 1A, Table I). There were no considerable differences in serum testosterone levels in the groups of testosterones and NaHS alone in comparison with the varicocele group (Figure 1A, Table I). However, the combined administration of testosterone and NaHS significantly increased serum testosterone levels compared to the varicocele group (p = 0.01, Figure 1A, Table I). The varicocele induction in rats markedly decreased testicular H_2_S levels compared with the sham group (p < 0.001, Figure 1B, Table I). There were no considerable differences in testicular H_2_S levels in the groups of testosterones and NaHS alone in comparison with the varicocele group (Figure 1B, Table I). However, the combined administration of testosterone and NaHS significantly increased testicular H_2_S levels compared with the varicocele group (p < 0.001, Figure 1B, Table I).

### Effects of subeffective doses of testosterone and NaHS alone or in combination on testicular histopathology

**Figure 2:**
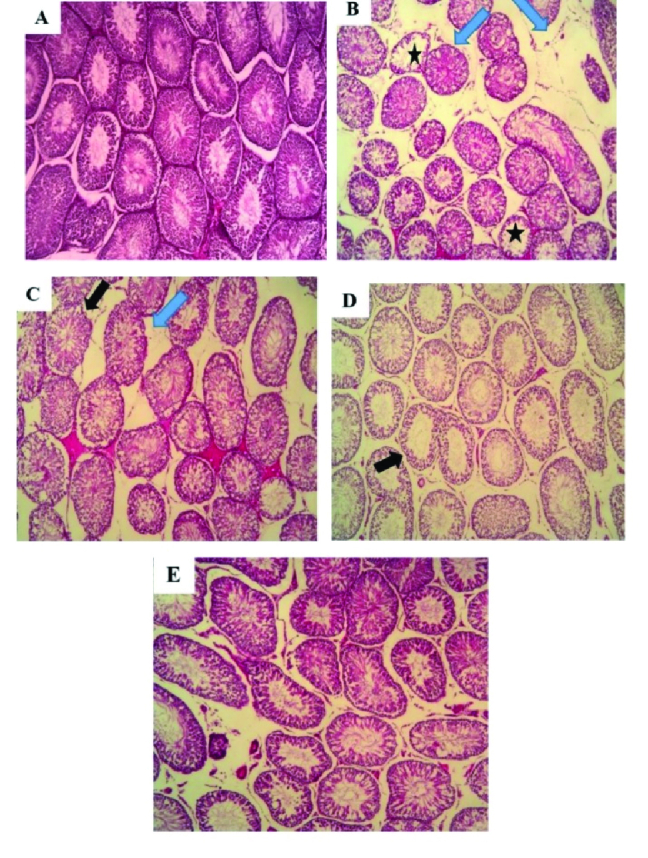
Changes in testicular histology in different groups with hematoxylin-eosin staining (light microscopy, × 400 magnification). (A) Sham, (B) Varicocele, (C) Testosterone, (D) NaHS, and (E) Testosterone + NaHS. Black arrows: the irregular architecture of the seminiferous tubules. Stars: Wider lumina of the seminiferous tubules. Blue arrows: the degeneration of the interstitial tissue and atrophy of the Leydig cells. Bar: 100 μm.

There was no detectable damage to the testicular tissues of the sham group (Figure 2A). The testicular tissues in the varicocele group showed severe changes in comparison with the sham group (Figure 2B). These changes include the irregular architecture and wider lumina of the seminiferous tubules and widening of the spaces between these tubules. The disorganized epithelium with changes in the basement membrane thickness, degeneration of the interstitial tissue, and atrophy of the Leydig cells were also seen. The combined but not individual administration of testosterone and NaHS considerably reduced the amount of histopathological damage (Figures 2C-E).

### Effects of subeffective doses of testosterone and NaHS alone or in combination on sperm count, motility, viability, and morphology

The varicocele induction in rats significantly decreased the number of spermatozoa compared to the sham group (p < 0.001, Table II). There were no considerable differences in the number of spermatozoa in the testosterone groups and NaHS alone compared with the varicocele group (Table II). However, the combined administration of testosterone and NaHS significantly increased the number of spermatozoa compared to the varicocele group (p < 0.001, Table II). The induction of varicocele in rats significantly decreased the percentage of progressively motile spermatozoa and increased the percentage of non-motile spermatozoa (both p < 0.001, Table II), but did not change the percentage of non-progressive motile spermatozoa (Table II) in comparison with the sham group. There were no considerable differences in these parameters in the groups of testosterones and NaHS alone compared to the varicocele group (Table II). However, the combined administration of testosterone and NaHS significantly increased the percentage of progressively motile spermatozoa and decreased the percentage of non-motile spermatozoa (p < 0.001 and p < 0.001, respectively), but did not change the percentage of non-progressive motile spermatozoa (Table II) compared to the varicocele group.

The varicocele induction in rats significantly decreased the percentage of viable spermatozoa compared to the sham group (p < 0.001, Table II). There were no considerable differences in the percentage of viable spermatozoa in the groups of testosterones and NaHS alone compared to the varicocele group (Table II). However, the combined administration of testosterone and NaHS remarkably enhanced the percentage of viable spermatozoa in comparison with the varicocele group (p < 0.001, Table II). The varicocele induction in rats significantly decreased the percentage of morphologically normal spermatozoa compared to the sham group (p < 0.001, Table II). There were no considerable differences in the percentage of morphologically normal spermatozoa in the groups of testosterones and NaHS alone compared to the varicocele group (Table II). However, the combined administration of testosterone and NaHS markedly enhanced the percentage of morphologically normal spermatozoa in comparison with the varicocele group (p = 0.01, Table II).

### Effects of subeffective doses of testosterone and NaHS alone or in combination on morphometric features of the seminiferous tubules and number of the Sertoli cells and spermatogonia

**Figure 3:**
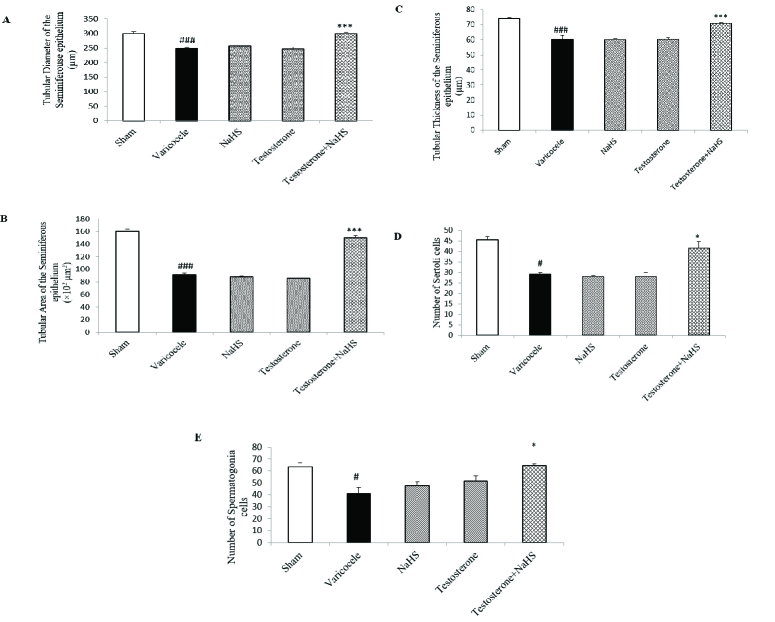
Changes in morphometric characteristics of the seminiferous tubules (A-C) and a number of the Sertoli cells (D) and spermatogonia (E) in different groups. A comparison among groups was performed by one-way analysis of variance (ANOVA) and Tukey's test. Data are expressed as Mean ± standard error of the mean (SEM). ${}^{\#}$ P < 0.05 vs. the sham group. ${}^{\#\#\#}$ P < 0.001 vs. the sham group. *P < 0.05 vs. the varicocele group. ***P < 0.001 vs. the varicocele group. NaHS: Sodium hydrosulfide.

The induction of varicocele in rats significantly decreased the tubular diameter and area, and thickness of the seminiferous epithelium of the seminiferous tubules in comparison with the sham group (all p < 0.001, Figures 3A-C, respectively, Table III). There were no considerable differences in these parameters in the groups of testosterones and NaHS alone compared to the varicocele group (Figures 3A-C, Table III). However, the combined administration of testosterone and NaHS significantly increased the tubular diameter and area, and thickness of the seminiferous epithelium of the seminiferous tubules in comparison with the varicocele group (all p < 0.001, Figures 3A-C, respectively, Table III). The varicocele induction in rats remarkably reduced the Sertoli cells and spermatogonia number compared to the sham group (p = 0.01 and p = 0.04, Figures 3D and E, respectively, Table III). There were no considerable differences in the number of the Sertoli cells and spermatogonia in the groups of testosterone and NaHS alone in comparison with the varicocele group (Figures 3D and E, respectively, Table III). However, the combined administration of testosterone and NaHS markedly enhanced the number of the Sertoli cells and spermatogonia in comparison with the varicocele group (both p = 0.03, Figures 3D and E, respectively, Table III).

### Effects of subeffective doses of testosterone and NaHS alone or in combination on MDA levels and SOD activity in the testicular tissues

**Figure 4:**
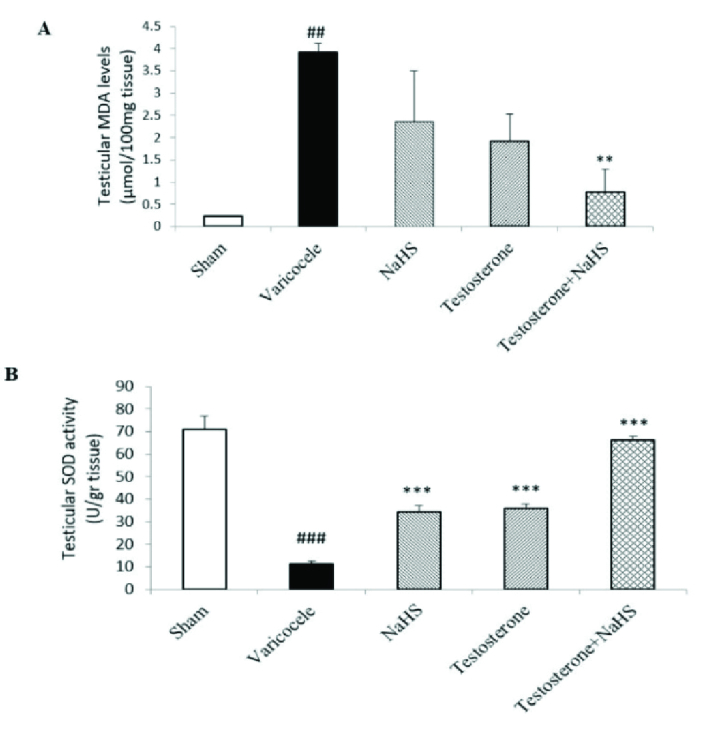
Changes in MDA levels (A) and SOD activity (B) in the testicular tissues in different groups. A comparison among groups was performed by one-way analysis of variance (ANOVA) and Tukey's test. Data are expressed as Mean ± standard error of the mean (SEM). ${}^{\#\#}$ P < 0.01 vs. the sham group. ${}^{\#\#\#}$ P < 0.001 vs. the sham group. **P < 0.01 vs. the varicocele group. ***P < 0.001 vs. the varicocele group. NaHS: Sodium hydrosulfide.

The varicocele induction in rats significantly increased testicular MDA levels compared to the sham group (p < 0.001, Figure 4A, Table IV). There were no considerable differences in testicular MDA levels in the groups of testosterone and NaHS alone than the varicocele group (Figure 4A, Table IV). However, the combined administration of testosterone and NaHS significantly decreased testicular MDA levels compared with the varicocele group (p < 0.001, Figure 4A, Table IV). The varicocele induction in rats significantly decreased testicular SOD activity compared to the sham group (p < 0.001, Figure 4B, Table IV). However, both the individual and combined administration of testosterone and NaHS significantly enhanced testicular SOD activity in comparison with the varicocele group (all p < 0.001, Figure 4B, Table IV).

### Effects of subeffective doses of testosterone and NaHS alone or in combination on the ratio of Bax to Bcl-2 protein expression in the testicular tissues

**Figure 5:**
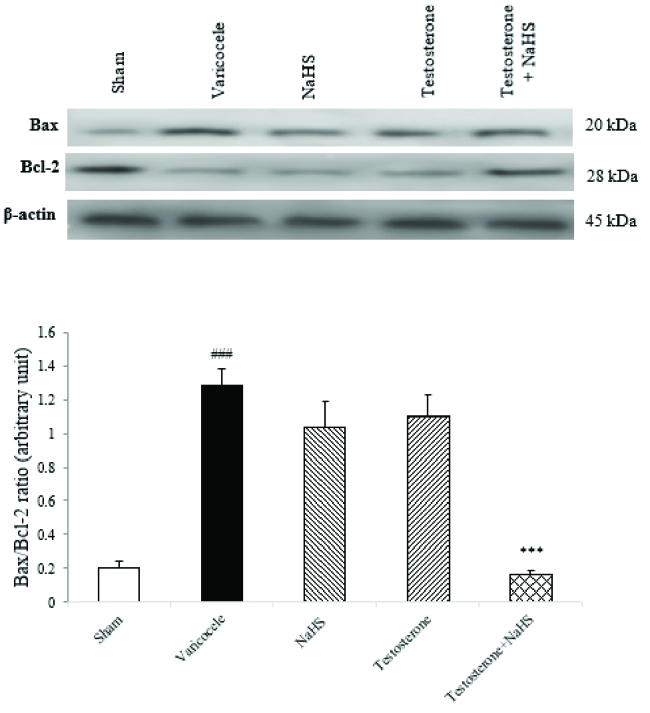
Changes in the Bax ratio to Bcl-2 protein expression in the testicular tissues in different groups. A comparison among groups was performed by one-way analysis of variance (ANOVA) and Tukey's test. Data are expressed as Mean ± standard error of the mean (SEM). ${}^{\#\#\#}$ P < 0.001 vs. the sham group. ***P < 0.001 vs. the varicocele group. NaHS: Sodium hydrosulfide, Bax: Bcl-2-associated X protein, Bcl-2: B-cell lymphoma 2.

The varicocele induction in rats considerably enhanced the ratio of Bax to Bcl-2 protein expression in the testicular tissues compared to the sham group (1.28 ± 0.08 vs. 0.20 ± 0.03, p < 0.001, Figure 5). There were no considerable differences in the ratio of Bax to Bcl-2 protein expression in the testicular tissues in the groups of testosterone (1.03 ± 0.13) and NaHS (1.19 ± 0.10) alone in comparison with the varicocele group (Figure 5). However, the combined administration of testosterone and NaHS significantly decreased the Bax to Bcl-2 protein expression ratio in the testicular tissues compared to the varicocele group (0.16 ± 0.01, 95% CI: 0.53-0.95, p < 0.001, Figure 5).

**Table 1:** Changes in serum testosterone and testicular H_2_S levels in different groups


[1.35in,lr]**Parameters** **Groups**	**Sham**	**Varicocele**	**NaHS**	**Testosterone**	**Testosterone + NaHS**	**95% confidence interval**
**Serum testosterone levels** ** ng/ml (n = 6)**	9.44 ± 1.80	3.30 ± 0.69 ${}^{\#}$	6.12 ± 1.02	4.91 ± 0.99	10.23 ± 0.95 ${}^{*}$	5.03-8.59
**Testicular H_2_S levels** **mM/gr tissue (n = 6)**	555.5 ± 17.88	465.51 ± 5.45	481.26 ± 8.37	475.39 ± 7.5	608.94 ± 21.09	493.66-542.12
Data are expressed as Mean ± standard error of the mean (SEM). A comparison among groups was performed by one-way analysis of variance (ANOVA) and Tukey's test. ${}^{\#}$ P < 0.05 vs. the sham group. *P < 0.05 vs. the varicocele group. NaHS: Sodium hydrosulfide, H_2_S: Hydrogen sulfide						

**Table 2:** Changes in sperm count, motility, viability, and morphology in different groups


[1.25in,ll]** Sperm parameters** **Functional**	**Sham**	**Varicocele**	**NaHS**	**Testosterone**	**Testosterone + NaHS**	**95% confidence interval**
**Sperm count (million/ml)**	165.83 ± 7.1	76.67 ± 3.02 ${}^{\#}$	84.17 ± 7.42	93.33 ± 9.08	164.67 ± 6.47 ${}^{*}$	100.71-133.16
**Progressive motility (%)**	33.5 ± 1.52	1.08 ± 0.72 ${}^{\#}$	0.08 ± 0.00	1.50 ± 0.87	5.66 ± 0.33 ${}^{**}$	13.24-3.48
**Nonprogressive motility (%)**	36.91 ± 1.76	43.58 ± 8.71	36.58 ± 5.20	30.47 ± 8.78	49.16 ± 9.35	32.62-46.04
**Nonmotile (%)**	28.75 ± 0.98	64.66 ± 4.91 ${}^{\#}$	63.16 ± 5.06	68.0 ± 8.72	23.33 ± 3.14 ${}^{*}$	40.99-58.17
**Viability (%)**	67.24 ± 2.28	48.00 ± 1.69 ${}^{\#}$	41.66 ± 2.81	48.83 ± 2.77	63.66 ± 2.66 ${}^{**}$	49.59-58.17
**Normal morphology (%)**	90.50 ± 2.7	73.33 ± 1.60 ${}^{\#}$	74.50 ± 2.04	75.33 ± 1.52	82.83 ± 1.30 ${}^{***}$	76.34-82.25
Data are expressed as Mean ± standard error of the mean (SEM). A comparison among groups was performed by one-way analysis of variance (ANOVA) and Tukey's test. ${}^{\#}$ P < 0.001 vs. the sham group. ***P < 0.05 vs. the varicocele group. **P < 0.01 vs. the varicocele group. *P < 0.001 vs. the varicocele group. NaHS: Sodium hydrosulfide						

**Table 3:** Changes in morphometric characteristics of the seminiferous tubules and a number of the Sertoli cells and spermatogonia in different groups


[1.3in,ll]**Parameters** **Groups**	**Sham**	**Varicocele**	**NaHS**	**Testosterone**	**Testosterone + NaHS**	**95% confidence interval**
**Diameter (µm)**	299.20 ± 6.33	248.71 ± 3.61 ${}^{\#\#\#}$	256.76 ± 3.83	246.45 ± 6.94	298.83 ± 5.42 ${}^{***}$	260.38-279.53
**Area ( × 10^2^ µm^2^)**	160 ± 3.35	91 ± 3.11 ${}^{\#\#\#}$	88 ± 1.22	86 ± 0.81	150 ± 3.87 ${}^{***}$	102.83-128.09
**Thickness (µm)**	74.13 ± 0.44	60.40 ± 2.51 ${}^{\#\#\#}$	60.02 ± 0.82	60.41 ± 0.84	70.93 ± 0.45 ${}^{***}$	62.79-67.45
**Number of Sertoli cells**	45.50 ± 1.50	29.00 ± 1.00 ${}^{\#}$	28.50 ± 0.50	28.00 ± 2.00	41.50 ± 3.50 ${}^{*}$	28.68-40.32
**Number of spermatogonia**	63.50 ± 3.50	41.00 ± 5.00 ${}^{\#}$	47.50 ± 3.50	51.50 ± 4.50	64.50 ± 1.50 ${}^{*}$	46.14-61.06
Data are expressed as Mean ± standard error of the mean (SEM). A comparison among groups was performed by one-way analysis of variance (ANOVA) and Tukey's test. ${}^{\#}$ P < 0.05 vs. the sham group. ${}^{\#\#\#}$ P < 0.001 vs. the sham group ${}^{*}$ P < 0.05 vs. the varicocele group. ${}^{***}$ P < 0.001 vs. the varicocele group. NaHS: Sodium hydrosulfide						

**Table 4:** Changes in MDA levels and SOD activity in the testicular tissues in different groups


[1.3in,ll]**Parameters** **Groups**	**Sham**	**Varicocele**	**NaHS**	**Testosterone**	**Testosterone + NaHS**	**95% confidence interval**
**MDA levels (µmol/100 mg** **tissue)**	0.24 ± 0.01	3.92 ± 0.19 ${}^{\#\#}$	2.35 ± 1.14	1.91 ± 0.61	0.77 ± 0.52 ${}^{**}$	1.14-2.54
**SOD activity (U/gr tissue)**	70.88 ± 5.96	11.43 ± 1.05 ${}^{\#\#\#}$	35.26 ± 2.57 ${}^{***}$	35.86 ± 2.05 ${}^{***}$	66.14 ± 1.56 ${}^{***}$	35.17-52.68
Data are expressed as Mean ± standard error of the mean (SEM). A comparison among groups was performed by one-way analysis of variance (ANOVA) and Tukey's test. ${}^{\#\#}$ P < 0.01 vs. the sham group. ${}^{\#\#\#}$ P < 0.001 vs. the sham group. ${}^{**}$ P < 0.01 vs. the varicocele group. ${}^{***}$ P < 0.001 vs. the varicocele group. NaHS: Sodium hydrosulfide, MDA: Malondialdehyde, SOD: Superoxide dismutase						

## 4. Discussion

In the current study, to the best of our knowledge for the first time, it was examined whether a subeffective dose of testosterone combined with a subeffective dose of H_2_S can improve varicocele-induced damages through a possible additive effect. As a starting point, we assessed the protective effects of the treatment regimen on the functional status of testicular tissues in varicocele rats since it is well known that varicocele causes testicular dysfunction (31). The first main function of the testes, steroidogenesis, is a multistep process by which cholesterol is converted to steroids such as testosterone (32). In this regard, preclinical and clinical studies have shown varicocele-induced impairment of steroidogenesis by a reduction in serum testosterone levels (33, 34). Similarly, this study observed a significant decrease in serum testosterone levels in rats subjected to varicocele. In addition, the subsequent histopathological examination of the testicular tissues confirmed this finding as it showed the Leydig cell atrophy in varicocele rats, indicating a reduction in testosterone-producing cells with varicocele induction. The second main function of the testes, spermatogenesis, is a process of multiple germ cell divisions to enhance their number and then differentiate into spermatozoa in the seminiferous tubules (35). Importantly, this process is highly dependent on testosterone, as it has been shown many times in the medical literature that hypospermatogenesis is associated with testosterone deficiency. Moreover, testosterone deficiency is reported to have detrimental effects on other sperm characteristics, including motility, viability, and morphology as well as morphometric features of the seminiferous tubules such as tubular diameter and thickness of the seminiferous epithelium (36). Accordingly, we found the deleterious effects of varicocele on these parameters along with decreased serum testosterone levels. However, from the therapeutic point of view, the combined administration of subeffective doses of testosterone and NaHS to varicocele rats reversed all the above pathological changes to the levels measured before varicocele induction, suggesting the improvement of testicular dysfunction.

In the next step, we evaluated the beneficial effects of the treatment regimen on testicular H_2_S levels in rats subjected to varicocele because it is thought that decreased H_2_S levels in testes contribute to varicocele-induced infertility (37). Our result showed a significant reduction in testicular H_2_S levels in varicocele, which was interestingly reversed by the combined administration of subeffective doses of testosterone and NaHS. Along with this, our histopathological examination of the testicular tissues revealed another interesting finding. In addition to the Leydig cell atrophy mentioned earlier, there was a considerable reduction in the number of Sertoli cells and spermatogonia in the testes of varicocele rats, which was markedly enhanced by the combined administration of subeffective doses of testosterone and NaHS. Importantly, in the male reproductive tract, H_2_S is mainly produced by cystathionine-β-synthase in the Leydig cells and cystathionine γ-lyase in the Sertoli cells and spermatogonia (13). Thus, our findings indicated the potential of this treatment regimen for protecting H_2_S-producing cells in testes and thereby maintaining normal testicular H_2_S levels.

After achieving such promising results from the treatment regimen, it was decided to elucidate some of the underlying molecular mechanisms. Varicocele-induced oxidative stress is well known to play a crucial role in damaging the male reproductive tract, including the testes (37). Oxidative stress is an imbalance between reactive oxygen species formation and their removal by antioxidant systems, leading to lipid peroxidation. Substantial evidence suggests that increased scrotal temperature due to retrograde blood flow to the pampiniform plexus is the most likely cause of oxidative stress induced by varicocele (38). In our study, the presence of oxidative stress, a significant increase in MDA levels is a major lipid peroxidation byproduct, and a decrease of SOD activity as an important antioxidant enzyme in the testicular tissues of varicocele rats were reported. From the therapeutic point of view, although the individual administration of subeffective doses of testosterone or NaHS to varicocele rats considerably enhanced testicular SOD activity, their co-administration provided better protection on lipid peroxidation and thus reduced oxidative stress in the testicular tissues.

In addition to its antioxidant effects, we evaluated the anti-apoptotic effects of the treatment regimen because it is indicated that varicocele-induced oxidative stress and apoptosis in the male reproductive tract are closely related processes (38). Although the mechanism is unknown, reactive oxygen species overproduction has been suggested to cause the efflux of cytochrome C from the mitochondria, which triggers signaling pathways for apoptosis, a type of cell death (39). In this terms, pro-apoptotic Bcl-2 family members (e.g., Bax), suppress the activity of anti-apoptotic Bcl-2 family members (e.g., Bcl-2) which is existing in the outer mitochondrial membrane, declaring that the ratio of pro- and anti-apoptotic Bcl-2 family members can be a good indicator to assess the occurrence of apoptosis (40). In this study, our findings showed a marked increase in the ratio of Bax to Bcl-2 protein expression levels in the testicular tissues of varicocele rats, which was remarkably reduced by the co-administration of subeffective doses of testosterone and NaHS.

The limitation of the present study was not evaluating the side effects of combined administration of subeffective doses of testosterone and NaHS in varicocele. Therefore, evaluating these side effects are suggested.

## 5. Conclusion

In the present study, the experimental varicocele caused decrease in the levels of serum testosterone and testicular H_2_S, as well as detrimental effects on sperm parameters and testicular histology. In contrast, the co-administration of subeffective doses of testosterone and the H_2_S donor NaHS, by reducing testicular oxidative stress and apoptosis through a possible additive effect, reversed all the above indicators to the levels measured before varicocele induction. Therefore, this study interestingly introduces a novel approach to benefit from the beneficial effects of testosterone therapy and decrease its side effects at the same time. Nevertheless, further preclinical investigations are needed to confirm the effectiveness of this therapeutic approach for clinical use.

##  Conflict of Interest

The authors declare no conflict of interest.
